# Genome characterisation of the first isolate of human enterovirus c99 from an acute flaccid paralysis case in Brazil

**DOI:** 10.1590/0074-02760240230

**Published:** 2025-06-27

**Authors:** Jéssica Tatiane Sauthier, Jéssica Barreto Dias, Cristiane de Sousa Ferreira, Brendo de Oliveira Nascimento Gomes, Ketlyn Araujo Fraga, Elisa Cavalcante Pereira, Bruna Mendonça da Silva, Letícia Ferreira Lima, Irving Martins da Silveira Gonçalves, Audrien Alves Andrade de Souza, Marília Alves Figueira de Melo, Alexandre Araujo Cunha dos Santos, Beatriz de Lima Alessio Müller, Aline dos Santos Moreira, Paola Cristina Resende, Eduardo de Mello Volotão, Edson Elias da Silva

**Affiliations:** 1 Fundação Oswaldo Cruz-Fiocruz, Instituto Oswaldo Cruz, Laboratório de Vírus Respiratórios, Exantemáticos, Enterovírus e Emergências Virais, Rio de Janeiro, RJ, Brasil; 2 Fundação Oswaldo Cruz-Fiocruz, Instituto Oswaldo Cruz, Laboratório de Genômica Aplicada e Bioinovações, Rio de Janeiro, RJ, Brasil; 3 Fundação Oswaldo Cruz-Fiocruz, Instituto Oswaldo Cruz, Laboratório de Avaliação e Promoção da Saúde Ambiental, Rio de Janeiro, RJ, Brasil; 4 Fundação Oswaldo Cruz-Fiocruz, Instituto Oswaldo Cruz, Rede de Plataformas Tecnológicas Fiocruz, Plataforma de Sequenciamento de Nova Geração, Rio de Janeiro, RJ, Brasil

**Keywords:** acute flaccid paralysis, human enterovirus C99, complete genome analysis

## Abstract

**BACKGROUND:**

Human enterovirus C99 (HEV-C99) is a member of the species Enterovirus C. Currently, three complete genomes of HEV-C99 were reported in Brazil, all obtained from children with gastroenteritis symptoms. Notwithstanding, no HEV-C99 complete genome associated with AFP cases in Brazil have been analysed so far.

**OBJECTIVES:**

In light of this, molecular characterisation of an HEV-C99 isolated from a case of acute flaccid paralysis (AFP) in Brazil was carried out.

**METHODS:**

In 2005, an HEV-C99 strain was isolated from a 2-year-old female child in Santa Catarina State, Brazil, showing classic symptoms of AFP. Stool sample was inoculated into specific cell cultures. Viral RNA was extracted, and polymerase chain reaction (PCR) were performed to amplify the VP1 gene; the sequence was analysed for molecular identification. Subsequently, the complete genome was sequenced and analysed, including a phylogenetic analysis of the VP1 gene.

**FINDINGS:**

The isolate, denominated HEV-C99/33322/BRA/2005 presented 85.85% identity to other HEV-C99 strains also described in Brazil, subsequently. Besides, the isolate grouped together with HEV-C99 cluster C strains. To our knowledge, this was the first described HEV-C99 isolated from an AFP case in Brazil.

**MAIN CONCLUSIONS:**

The data generated in this study bolster the role of HEV-C99 as an etiologic agent of AFP. Furthermore, this research enhances our knowledge regarding the HEV-C99 genetic diversity.

Human enteroviruses (HEV) are members of the genus *Enterovirus* within the *Picornavirales* order, *Picornaviridae* family. They are categorised into four species: *Enterovirus A*, *B*, *C*, and *D*. *Enterovirus C* species is particularly noteworthy for its association with various clinical disorders, including common cold, acute haemorrhagic conjunctivitis, acute flaccid paralysis (Poliomyelitis, AFP), aseptic meningitis, and encephalitis. This species includes 23 serotypes, such as the three poliovirus (PV1, PV2, and PV3), group A coxsackievirus (CVA-1, CVA-11, CVA-13, CVA-17, CVA-19-22, CVA-24), as well as HEV-C95, HEV-C96, HEV-C99, HEV-C102, HEV-C104-C105, HEV-C109, HEV-C113, and HEV-C116-C118.^1^ ^,^ ^2^

Enteroviruses are non-enveloped viruses, with a single-stranded RNA genome of approximately 7.4-7.5 kb in size. The viral particle contains 60 copies of four capsid proteins: VP4, VP2, VP3, and VP1. The viral RNA comprises a long open reading frame (ORF) flanked by 5′ and 3′ untranslated regions (UTR). The 5′-UTR (approximately 740-750 nucleotides in length), contains important structural elements such as a cloverleaf structure and an internal ribosome entry site (IRES), involved in the replication of viral RNA. The viral RNA is translated into a single polyprotein, which is subsequently cleaved into three polyprotein precursors, denoted P1, P2, and P3. P1 is the precursor of the structural proteins (VP1-to-VP4), while P2 and P3 are precursors of the non-structural proteins 2A-to-2C and 3A-to-3D, respectively.^3^ ^,^ ^4^

HEV-C99 is a member of *Enterovirus C* species and was first isolated in Bangladesh, in the year 2000.^5^ After that, many other HEV-C99 strains have been isolated worldwide, including Brazil, Spain, Madagascar, China, Kenya, India, Oman and Philippines.^5^ ^-^ ^12^ HEV-C99 consists of three genetic clusters, denominated A, B and C, and homotypic strains exhibited nucleotide or amino acid identity above the cutoff values of 75.0% and 88.0%, respectively.^13^ ^,^ ^14^ ^,^ ^15^ Currently, three complete genomes of HEV-C99 were reported in Brazil, all obtained from children with gastroenteritis symptoms.^6^ ^,^ ^7^ Notwithstanding, no HEV-C99 complete genome associated with AFP cases in Brazil has been analysed so far. Herein, we described the complete genome sequence of an HEV-C99 isolated from a patient with AFP symptoms in Brazil during the year of 2005 as well as its genetic relationship with other HEV-C99 isolates.

## SUBJECTS AND METHODS

*Ethics statements* - The sample was collected in the scope of the Global Poliovirus Surveillance Program, as part of the World Health Organization (WHO) and National Public Health responses. This work was submitted to and approved by the Ethics Committee (Fiocruz/IOC) under number 4.894.43 in compliance with the Declaration of Helsinki (1975).

*Virus isolation* - HEV-C99/33322/BRA/2005 was isolated in 2005 from a 2-year-old female child in the State of Santa Catarina, Brazil. The patient presented classical AFP (Poliomyelitis) symptoms. Stool sample was collected by the Brazilian National Public Health Network 12 days after the beginning of the symptoms and sent to our laboratory, a WHO Regional Reference Laboratory (RRL) and National Reference Laboratory for the Ministry of Health of Brazil. Faecal specimen was processed according to the WHO standard procedures.^16^ Chloroform-treated stool suspension, in volumes of 200 µL were inoculated into three types of polio/enterovirus-sensitive cell culture - RD (human rhabdomyosarcoma), L20B (mouse L cells transfected with the gene for the human cellular receptor for poliovirus) and Hep2C (human cervix carcinoma). The individual isolate was named according to the following convention: HEV followed by the number denoting the type/isolate number/3-letter country followed by the year of isolation (*e.g.*, HEV-C99/33322/BRA/2005). For the purpose of this study, the HEV-C99 isolate name has been abbreviated to 33322.

*Primary characterisation of HEV-C99* - Viral RNA was extracted from an aliquot of 140 µL of RD cell culture supernatant using QIAamp Viral RNA Mini Kit (Qiagen, Hilden, Germany) and stored at -80ºC for further analysis. cDNA was prepared from 9 µL of stock viral RNA using 1 µL of Superscript II Reverse Transcriptase (Thermo Fisher Scientific, Carlsbad, CA, USA). Polymerase chain reactions (PCR) were performed to amplify the VP1 gene using primer sets AN88/AN89 - 700 base pairs (bp) - in the first reaction, and 222/292 - 350 bp - in the second reaction.^17^ PCR products were purified using QIAquick Gel Extraction Kit (Qiagen, Hilden, Germany), and cycle sequencing reactions were carried out using BigDye terminator chemistry (Applied Biosystems, Carlsbad, CA, USA) on an ABI 3130XL instrument. The resulting sequences, corresponding to the VP1 gene, were analysed using BLASTn/BLASTx against the GenBank database and molecular typed as HEV-C99.

*Molecular detection and sequencing* - To obtain the complete genome sequence, four long PCR fragments were performed using GoTaq Long PCR Master Mix (Thermo Fisher Scientific, Carlsbad, CA, USA) using a primer set described by Bessaud and collaborators.^18^ PCR mixture for each reaction consisted of 25 µL of GoTaq Long PCR Master Mix (Thermo Fisher Scientific, Carlsbad, CA, USA), 1 µL of each primer (50 pmol) and 18 µL of RNAse free water (Promega, Madison, WI, USA) resulting a final volume of 50 µL. The cycling parameters for amplifying the four fragments were as follows: an initial denaturation at 95ºC for 2 min, followed by 35 cycles of 94ºC for 20 s, 50ºC for 30 s and 65ºC for 1 min, with a final extension at 72ºC for 10 min. The four amplified products were analysed on a 1% agarose gel and visualised on a UV light transilluminator. Overlapping products were purified using QIAquick Gel Extraction Kit (Qiagen, Hilden, Germany) following the manufacturer’s instructions and the DNA concentration was then assessed by spectrophotometry using NanoDrop™ (Thermo Fisher Scientific, Carlsbad, CA, USA). DNA fragment libraries were prepared using the Illumina DNAprep Preparation kit (Illumina, USA) and sequenced on an Illumina MiSeq System (Illumina, USA) with an Illumina micro v2 reagent kit (2 x 150 paired end reads).

*Bioinformatic analysis* - The quality of the read libraries from the DNA sequencing was accessed using FastQC^19^ and trimmed based on a Phred quality score cut-off of 20.^20^ The trimmed read libraries were then assembled into contigs with MetaSPAdes.^21^ To confirm the viral origin of the contigs, the libraries were mapped against the nonredundant (nr) database. Contigs related to HEV were compared to sequences in GenBank nucleotide and protein databases using BLASTn/BLASTx.^22^ For alignments, ORF prediction and genome annotation, the Geneious Prime 2023 software (https://www.geneious.com) was used.

*Phylogenetic analysis of VP1 gene* - Complete VP1 ORF of HEV-C99 sequences were aligned with MAFFT (https://www.geneious.com).^23^ Phylogenetic inference was conducted with IQ-TREE web server,^24^ where a tree was generated using the best-fit model selected by MEGA X and the maximum likelihood method.^25^ The General Time Reversible model^26^ with gamma distribution and invariant sites (GTR+G+I) was applied, along with 1,000 nonparametric bootstrap replicates for statistical support.

## RESULTS

*Primary classification of HEV-C99* - The isolate 33322 was obtained from a 2-year-old female child showing classical presentation of AFP in the State of Santa Catarina, Brazil, in 2005. Conventional PCR followed by Sanger sequencing confirmed it as an HEV-C99. Comparison with GenBank sequences revealed that the closest relative in the capsid region/VP1 sequence was the human enterovirus C99 isolate 3291/BRA-PA/10 (GenBank accession number MH484164). This isolate shows 85.27% nucleotide identity and 93.49% amino acid similarity with 33322 isolate and was isolated from a gastroenteritis case during 2010 in Brazil.

*Overview of high-throughput sequencing (HTS)* - HTS generated 494,034 raw reads, with the number of high-quality paired-end reads closely related to HEV ranging between 29,772 and 302,016. These reads were assembled de novo into 1,847 distinct contigs using the MetaSPAdes assembler, of which four were related to HEV resulting in the complete genome.

*Complete genome of isolate HEV-C99/33322/BRA/2005* - The assembled genome of the strain 33322 has a GC content of 45.2% and a length of 7,456 nucleotides (nt). It contains a single ORF that encodes a large viral polyprotein of 2,211 amino acids. The 5’ and 3’ UTRs are 755 nt and 68 nt long, respectively. Isolate 33322 is most closely related to human enterovirus C99 isolate 3291/BRA-PA/10 (GenBank accession number MH484164), showing 85.85% nucleotide and 95.75% amino acid identity at the VP1 gene. The genome is predicted to contain a long coding sequence that encodes four structural genes (VP1, VP2, VP3, and VP4) and seven non-structural genes (2A, 2B, 2C, 3A, 3B, 3C, and 3D) (Table). The complete genome sequence was deposited in GenBank (accession number PP497091).

**TABLE:** Analysis of nucleotide and amino acid identity of isolate 33322

Genome region	Nucleotide			Amino acid		
	Length	% identity	Best hit (GenBank accession number)	Length	% identity	Best hit (GenBank accession number)
5’UTR	755	93.70	Enterovirus C99 isolate 3291/BRA-PA/10 (MH484164)	NA	NA	NA
P1 region						
VP4	180	89.44	Enterovirus C99 isolate BRA/TO-16 (MK689071)	60	100	Enterovirus Ningbo CHN/171/2009 (AFK93160)
VP2	852	81.22	Enterovirus C99 isolate BRA/TO-16 (MK689071)	284	96.83	Enterovirus C99 HEV-99_68229 (AEO51787)
VP3	612	86.23	Enterovirus C99 isolate 3944/BRA-PA/11 (MH484166)	204	88.70	Enterovirus C99 ADA-18-059-005 (QRG33061)
VP1	876	85.27	Enterovirus C99 isolate 3291/BRA-PA/10 (MH484164)	292	93.49	Enterovirus C99 isolate 3291/BRA-PA/10 (QCP69018)
P2 region						
2A	564	85.64	Enterovirus C99 isolate 3944/BRA-PA/11 (MH484166)	188	93.62	Enterovirus C99 isolate 3291/BRA-PA/10 (QCP69018)
2B	99	89.80	Enterovirus C99 isolate 3291/BRA-PA/10 (MH484164)	33	100	Enterovirus C99 isolate 3291/BRA-PA/10 (QCP69018)
2C	1170	86.50	Enterovirus C99 isolate 3291/BRA-PA/10 (MH484164)	390	97.69	Enterovirus C99 isolate 3944/BRA-PA/11 (QCP69020)
P3 region						
3A	252	89.88	Enterovirus C99 isolate 3944/BRA-PA/11 (MH484166)	84	98.72	Enterovirus C99 isolate 3291/BRA-PA/10 (QCP69018)
3B	120	89.08	Enterovirus C99 isolate 3291/BRA-PA/10 (MH484164)	40	100	Enterovirus C99 isolateVRM9D/UFS-NGS/ZAF/2022 (XBP46903)
3C	537	89.20	Enterovirus C99 isolate 3944/BRA-PA/11 (MH484166)	179	89.39	Coxsackievirus A24 (ALB77613)
3D	1371	85.63	Poliovirus 1 RUS37255 (KC880378)	457	98.03	Enterovirus C99 isolate VRM9D/UFS-NGS/ZAF/2022 (XBP46903)
		85.35	Enterovirus C99 isolate BRA/TO-16 (MK689071)			
3’UTR	68	100	Enterovirus C strain Polozj-3 (MZ546188)	NA	NA	NA
Entire genome	7456	85.85	Enterovirus C99 isolate 3291/BRA-PA/10 (MH484164)	2211	95.75	Enterovirus C99 isolate 3291/BRA-PA/10 (QCP69018)

NA: not applies.

*VP1 phylogenetic analysis* - A phylogenetic tree was reconstructed using alignments based on the complete nucleotide sequence of the VP1 gene, including representative sequences from other HEV-C99 isolates [Supplementary data (Table)]. HEV-C99 is known to consist of three clusters, denominated A, B and C.^14^ Bootstrap values for these clusters were 94%, 99%, and 83%, respectively, in this study. The analysis revealed that isolate 33322, as well the other Brazilian HEV-C99 strains, clustered within the cluster C, with a bootstrap value of 83%. The VP1 sequence positioned isolate 33322 among HEV-C99 strains subsequently reported in Brazil (Figure). In comparison to the complete genomes also described in Brazil, isolate 33322 showed 85.85% nucleotide and 95.75% amino acid identity to enterovirus C99 isolate 3291/BRA-PA/10 (GenBank accession number MH484164); 85.56% nucleotide and 95.34% amino acid identity to enterovirus C99 isolate 3944/BRA-PA/11 (GenBank accession number MH484166); and 83.91% nucleotide and 95.11% amino acid identity to enterovirus C99 isolate BRA/TO-16 (GenBank accession number MK689071), thus supporting its typing as HEV-C99. Previous studies reported that homotypic strains exhibited nucleotide or amino acid identity above the cut-off values of 75.0% and 88.0%, respectively.^13^ ^,^ ^14^ ^,^ ^15^ Curiously, two sequences from United States isolates branched separately from all previously reported HEV-C99 clusters (bootstrap value of 100%) and may represent a putative HEV-C99 distinct genetic lineage.

**Figure:**
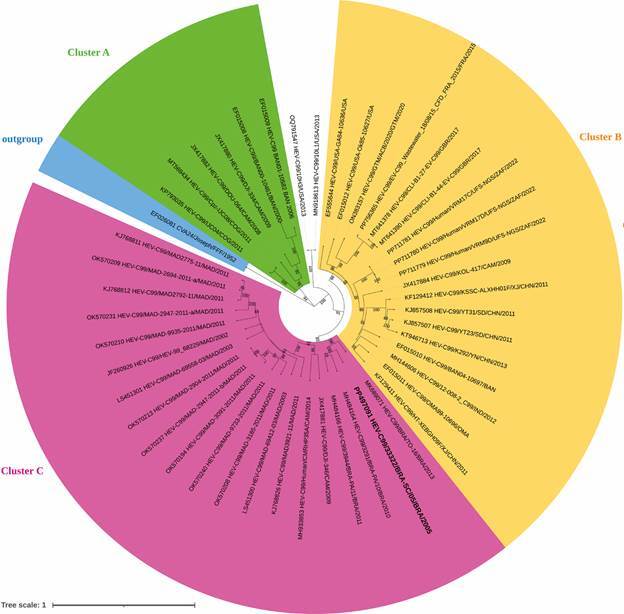
Molecular phylogenetic analysis of human enterovirus C99 based on complete VP1 gene nucleotide sequence. Numbers at internal nodes represent the bootstrap support values (percentages) determined for 1,000 replications. For clarity, most bootstrap values of less than 50% have been omitted. The analysis involved 47 nucleotide sequences, and a total of 926 positions included in the final dataset. The GenBank accession numbers, country, and year of isolation for each reference isolate are indicated, if known (BAN, Bangladesh; BRA, Brazil; CAM, Cameroon; COG, Republic of the Congo; CHN, China; FFF, Africa; FRA, France; GBR, United Kingdom; GTM, Guatemala; IND, India; MAD, Madagascar; OMN, Oman; USA, United States; ZAF, South Africa). The sequence from this study is represented in boldface type.

## DISCUSSION

This study aimed to assess the genome characterisation of an HEV-C99 isolate from a child with classical presentation of AFP in Brazil during 2005. Currently, only 28 complete genomes of HEV-C99 are available in the GenBank database.^27^ These genomes have been identified worldwide, including in Africa,^28^ ^,^ ^29^ ^,^ ^30^ ^,^ ^31^ Asia,^5^ ^,^ ^9^ ^,^ ^10^ ^,^ ^11^ ^,^ ^12^ Europa,^8^ ^,^ ^32^ North America, and South America.^6^ ^,^ ^7^ In the present study, a complete genome of HEV-C99 was analysed, adding to the collection of HEV sequences in the GenBank database. Due to the global polio eradication program, non-polio enteroviruses (NPEV), such as HEV-C99, have played a critical role in association with AFP.^8^ ^,^ ^33^ ^,^ ^34^

The HEV-C99 is an emerging type of HEV-C associated with AFP cases and was first isolated in Bangladesh in 2000;^5^ however, no complete genome sequences were obtained from South America before 2019. To the best of our knowledge, three complete HEV-C99 isolates have been described in Brazil, all from children with classical gastroenteritis symptoms.^6^ ^,^ ^7^ Phylogenetic analysis of isolate 33322, based on the VP1 nucleotide sequence, revealed that the strain is closely related to strains isolated in Brazil from children exhibiting symptoms of gastroenteritis.^6^ ^,^ ^7^ This highlights the global distribution of HEV-C99, as well as its role in AFP, gastroenteritis, and asymptomatic infections.^6^ ^,^ ^7^ ^,^ ^31^ ^,^ ^35^

HEV-C99 and coxsackievirus A24 (CVA-24) are closely related viruses, both classified within the *Enterovirus C* species. Initially, at the time of its isolation, isolate 33322 was molecularly typed as CVA-24. Until 2001, CVA-24 had never been associated with AFP, but it had been linked to respiratory infections, Hand-Foot-and-Mouth disease (HFMD), and infantile gastroenteritis.^36^ Recently, documented cases have shown that CVA-24 infection can lead to neurological complications, including AFP.^37^ ^,^ ^38^ The diversity in disease manifestations associated with CVA-24 has been proposed to result from profound biological effects of certain mutations that do not affect the phylogenetic positions of the virus strains.^39^ In Brazil, complete genomes of HEV-C99 have only been described from gastroenteritis cases.^6^ ^,^ ^7^ Nevertheless, they have also been detected in stool samples from AFP patients in Spain, Nigeria, and Senegal, as well as in Brazil during the Global Polio Eradication Initiative.^8^ ^,^ ^33^ ^,^ ^34^ ^,^ ^35^

NPEV isolated during AFP surveillance are often not thoroughly characterised at the molecular level. This lack of molecular characterisation creates a gap in enterovirus surveillance, as understanding the genetic variability of NPEV is essential for gaining insights into the molecular epidemiology of viral infections. Molecular characterisation of HEV-C99 genomes can provide valuable information about the viral diversity circulating in a population. Furthermore, in the context of poliovirus eradication efforts, surveillance of NPEV is crucial for understanding the prevalence, transmission dynamics, and clinical outcomes associated with HEV-C99 infections in different populations and settings. This knowledge is essential for public health efforts aimed at managing acute outbreaks and potentially integrating these findings into broader disease surveillance and control strategies.

Our findings highlight the detection of HEV-C99 in an AFP case in Brazil and emphasise the importance of improving our understanding of the epidemiology of AFP in areas where wild polioviruses have been eliminated.

## Data Availability

Sequence data that support the findings of this article have been deposited in GenBank with the accession code PP497091
