# Supplementary material for: Genome characterisation of the first isolate of human enterovirus c99 from an acute flaccid paralysis case in Brazil
Source: Mem Inst Oswaldo Cruz. 2025 Jun 27;120:e240230. doi: 10.1590/0074-02760240230 (PMC12208675; doi:10.1590/0074-02760240230)
Supplement: Supplementary file 1 [file 1678-8060-mioc-120-e240230-s.pdf]

TABLE

GenBank accession numbers of complete VP1 sequences utilised in the phylogenetic analysis

| Sequence identification | GenBank accession number |
|---|---|
| CVA24/Joseph/FFF/1952 | EF026081 |
| HEV-C99/MAD-2694-2011-a/MAD/2011 | OK570209 |
| HEV-C99/MAD2792-11/MAD/2011 | KJ768812 |
| HEV-C99/MAD-2947-2011-a/MAD/2011 | OK570231 |
| HEV-C99/MAD-9935-2011/MAD/2011 | OK570210 |
| HEV-C99/HEV-99_68229/MAD/2002 | JF260926 |
| HEV-C99/MAD-69558-03/MAD/2003 | LS451301 |
| HEV-C99/MAD-2904-2011/MAD/2011 | OK570213 |
| HEV-C99/MAD-2947-2011-b/MAD/2011 | OK570237 |
| HEV-C99/MAD-3091-2011/MAD/2011 | OK570194 |
| HEV-C99/MAD-9733-2011/MAD/2011 | OK570240 |
| HEV-C99/MAD-3185-2011/MAD/2011 | OK570208 |
| HEV-C99/MAD-69412-03/MAD/2003 | LS451300 |
| HEV-C99/MAD3921-11/MAD/2011 | KJ768826 |
| HEV-C99/Human/CMRHP35A/CMR/2014 | MH933853 |
| HEV-C99/DJI-346/CAM/2009 | JX417881 |
| HEV-C99/3944/BRA-PA/11/BRA/2011 | MH484166 |
| HEV-C99/3291/BRA-PA/10/BRA/2010 | MH484164 |
| HEV-C99/33322/BRA-SC/05/BRA/2005 | PP497091 |
| HEV-C99/BRA/TO-16/BRA/2013 | MK689071 |
| HEV-C99/HT-XEBGH09F/XJ/CHN/2011 | KF129411 |
| HEV-C99/OMA99-10696/OMA | EF015011 |
| HEV-C99/12-008-2_C99/IND/2012 | MH144606 |
| HEV-C99/BAN04-10697/BAN | EF015010 |
| HEV-C99/K292/YN/CHN/2013 | KT946713 |
| HEV-C99/YT23/SD/CHN/2011 | KJ857507 |
| HEV-C99/YT31/SD/CHN/2011 | KJ857508 |
| HEV-C99/KSSC-ALXHH01F/XJ/CHN/2011 | KF129412 |
| HEV-C99/KOL-417/CAM/2009 | JX417884 |
| HEV-C99/Human/VRM9D/UFS-NGS/ZAF/2022 | PP711779 |
| HEV-C99/Human/VRM17D/UFS-NGS/ZAF/2022 | PP711780 |
| HEV-C99/Human/VRM17C/UFS-NGS/ZAF/2022 | PP711781 |
| HEV-C99/CLI-B1-44-EV-C99/GBR/2017 | MT641390 |
| HEV-C99/CLI-B1-27-EV-C99/GBR/2017 | MT641378 |
| HEV-C99/EV-C99_Wastewater_18/08/15_CFD_FRA_2015/FRA/2015 | PP756365 |
| HEV-C99/GTM/ACB/2020/GTM/2020 | ON383157 |
| HEV-C99/USA-Ok85-10627/USA | EF015012 |
| HEV-C99/USA-GA84-10636/USA | EF555644 |
| HEV-C99/10L1/USA/2013 | MN918613 |
| HEV-C99/10H3/USA/2013 | OQ791547 |
